# Cardiorespiratory signatures of necrotizing enterocolitis: a 4-NICU study of very low birth weight infants

**DOI:** 10.1038/s41390-025-04631-8

**Published:** 2025-12-16

**Authors:** Sherry L. Kausch, Zachary A. Vesoulis, Colm P. Travers, Carie Taveras, Rachel Benz, Amanda Duncan, Angela K. S. Gummadi, Katy N. Krahn, Rakesh Sahni, Joseph Isler, Namasivayam Ambalavanan, J. Randall Moorman, Douglas E. Lake, Karen D. Fairchild, Brynne A. Sullivan

**Affiliations:** 1 Department of Pediatrics, University of Virginia School of Medicine, Charlottesville, VA USA; 2 Department of Pediatrics, Washington University in St. Louis School of Medicine, St. Louis, MO USA; 3 Department of Pediatrics, University of Alabama at Birmingham, Birmingham, AL USA; 4 Department of Pediatrics, Columbia University Vagelos College of Physicians and Surgeons, New York, NY USA; 5 Department of Medicine, University of Virginia School of Medicine, Charlottesville, VA USA

## Abstract

**Background:**

Necrotizing enterocolitis (NEC) is a disorder in very low birth weight (VLBW, <1500 g) infants, associated with significant morbidity and mortality. Earlier detection of NEC may improve outcomes. We hypothesized that NEC’s physiological signature resembles that of sepsis and that our model developed for sepsis, the Pulse Oximetry Warning System (POWS), would also predict NEC.

**Objectives:**

Assess relationships between continuous heart rate (HR) and oxygen saturation (SpO_2_) features and NEC risk, and evaluate POWS performance for predicting medical and surgical NEC.

**Methods:**

In a retrospective multicenter analysis, we studied VLBW infants at four level IV NICUs, identifying cases of late-onset sepsis (LOS) and NEC (Bell stages 2 or 3). Using HR and SpO_2_ data, we calculated features and POWS scores and assessed associations with NEC and LOS risk.

**Results:**

Among 3914 infants evaluated, 5% had NEC and 13% had LOS. Cardiorespiratory data analysis showed that HR/SpO_2_ patterns had similar risk associations with NEC and LOS. POWS AUC was 0.758 for NEC, 0.804 for sepsis, 0.791 for NEC or LOS, and 0.808 for NEC requiring surgery.

**Conclusion:**

NEC shares a cardiorespiratory signature with LOS. POWS predicted NEC with a dynamic rise before clinical diagnosis.

**Impact:**

- A cardiorespiratory early warning score analyzing heart rate and oxygen saturation, trained to predict late-onset sepsis within 24h across, predicts necrotizing enterocolitis.
- Heart rate and oxygen saturation features had the same patterns of association with necrotizing enterocolitis as sepsis.
- The results increase understanding of physiologic signatures of neonatal sepsis and necrotizing enterocolitis

## Introduction

Although the incidence of necrotizing enterocolitis (NEC) has declined in recent years for very low birth weight (VLBW, <1500 g) infants,^1^ NEC continues to be a major cause of morbidity and mortality.^2–4^ Earlier diagnosis and treatment of NEC could lead to improved outcomes, and one promising avenue for this is quantitative prediction of NEC risk using routinely collected continuous vital signs.

Cardiorespiratory changes start in the early phase of a systemic inflammatory response. Our group previously developed a heart rate characteristics index (HeRO score) as an early warning system for sepsis.^5^ Sepsis and NEC are strong inducers of systemic inflammation, a pathologic commonality that can be leveraged to detect multiple conditions with a single tool. We have previously shown that abnormal heart rate patterns occur in the preclinical stage of NEC, hours before obvious clinical deterioration.^6^ In subsequent work on sepsis prediction, we added analysis of oxygenation patterns to heart rate pattern analysis to capture an additional domain of illness, as respiratory instability commonly occurs in preterm infants during acute inflammatory illnesses. We developed a pulse oximetry warning system (POWS) to analyze continuous heart rate (HR) and oxygen saturation (SpO_2_) data and showed that it predicted the diagnosis of blood culture-positive sepsis in the next 24 h.^7,8^ In this study, we sought to extend our previous work on POWS to explicitly evaluate POWS performance in detecting NEC. We hypothesized that the signatures of illness observed in HR and SpO_2_ patterns around sepsis would also occur before clinical deterioration due to NEC, even in the absence of bacteremia.

Using a large, multicenter dataset, our aims were to (1) examine the association of HR and SpO_2_ features with NEC risk and compare those to patterns previously identified to be associated with sepsis, (2) evaluate the performance of POWS in predicting NEC, and (3) evaluate change in POWS in the preclinical phase of medical and surgical NEC.

## Methods

### Study population

We studied VLBW infants admitted to four NICUs: University of Virginia Children’s Hospital (2012–2023), Morgan Stanley Children’s Hospital of New York, Columbia University (2012–2019), St. Louis Children’s Hospital, Washington University School of Medicine (2016–2021), and University of Alabama at Birmingham (2018–2023). The participating NICUs are part of an NICHD-funded observational research study, “Predictive Informatics Monitoring in the Neonatal Intensive Care Unit” (HD072071), which involves the collection of continuous bedside monitor vital sign data from VLBW infants.

The study was approved by the Institutional Review Board at each center with waiver of informed consent. We excluded infants with major chromosomal or congenital anomalies and infants who died within three days of birth. Clinical data were recorded in a REDCap database and consisted of demographic and clinical variables, including late-onset sepsis (LOS) and NEC events.

### Sepsis and NEC definition

Clinicians at each site reviewed events in which a blood culture was obtained after 72 h and before 120 days of age in VLBW infants with a clinical suspicion of illness and who were not on antibiotics in the preceding 2 days. We classified the event as LOS if the blood culture yielded a pathogen and the infant was treated with at least five days of antibiotics.

We classified an event as NEC if it met Bell’s criteria for stage 2 or 3 NEC,^9^ including clinical signs of illness, radiographic evidence of pneumatosis, portal venous gas, or pneumoperitoneum, a blood culture was obtained to evaluate for concurrent bacteremia, and antibiotics were administered for at least five days. NEC cases were designated as surgical if the infant had an exploratory laparotomy or peritoneal drain placement after the initial diagnosis. NEC cases treated with antibiotics and bowel rest were designated as medical. Events documented in the medical record as focal or spontaneous intestinal perforation without bacteremia were excluded.

The time of diagnosis for LOS and NEC was defined as the time the blood culture was ordered. To evaluate differences in clinical variables between LOS and NEC, we used clinical and demographic data entered in a multicenter REDCap database. To evaluate the relationship between cardiorespiratory features and LOS or NEC, we included only infants who had archived HR and SpO_2_ data available. For infants with more than one episode of LOS or NEC, only the first episode was included as an event in the analyses. For infants with both LOS and non-concurrent NEC (diagnosis greater than 10 days apart), the first episode of each disorder was included as an event. Every hour window of data was labeled as “control,” “event,” or censored. Windows in the 24-h period preceding the time of positive blood culture were labeled “event”, those falling in the seven days following events were censored, and all other windows falling between 72 h after birth and PNA 120 were labeled as control.

### HR and SpO2 data collection and preprocessing

Continuous HR and SpO_2_ data were collected from standard NICU bedside monitors (GE, Philips) and archived using the BedMaster system (Hillrom’s Medical Device Integration Solution, Chicago, IL). Electrocardiogram-derived HR and pulse oximeter-derived SpO_2_ were collected at 0.5 Hz at two centers. Two centers collected data at 1 Hz, and were down-sampled to 0.5 Hz to match the other sites. SpO_2_ was measured with the default averaging time of 8 s. HR and SpO_2_ data underwent single-step preprocessing to remove values representing incontrovertible artifact (zeros). Times with missing vital sign data were excluded from analysis.

In 10-min non-overlapping windows, we calculated HR and SpO_2_ mean, standard deviation (SD), skewness, kurtosis, and maximum cross-correlation (XCORR) of HR-SpO_2_. XCORR reports on co-trending of the two signals.^10^ The MATLAB XCORR function was used with a lag time of 30 s. In prior work, we showed that an increased XCORR HR-SpO_2_ was associated with apnea and periodic breathing.^11^ Skewness is a measure of symmetry; symmetric distributions have a value of 0, positive values for distributions with a longer tail to the right, and negative values for distributions with a longer tail to the left. Kurtosis is a measure of dispersion, measuring how the data disperse between the center of a distribution and the tails. Distributions that are symmetric and Gaussian have a value of three. Values greater than three indicate heavier tails while values less than three indicate fewer extreme observations.

### Pulse Oximetry Warning System (POWS) score calculation

We used seven of the nine calculated HR and SpO_2_ features described above as predictors in our previously published POWS model. The POWS model was trained on data from the University of Virginia Children’s Hospital NICU. Model performance was tested using data from two additional NICUs: Morgan Stanley Children’s Hospital of New York and St. Louis Children’s Hospital. The model was trained to predict LOS and includes 3 HR features (SD, skewness, and kurtosis), 3 SpO_2_ features (mean, skewness, and kurtosis) and the XCORR of HR and SpO_2_.^8^ The model uses the 10-min features as inputs and calculates an hourly POWS risk score for the duration of the NICU admission for all VLBW infants when data were available. The score was developed and validated to represent the fold-increased risk of LOS diagnosis in the next 24 h compared to the baseline sepsis risk for all VLBW infants in our cohort at all times. For example, a POWS value of 3 indicates a VLBW infant has a 3-fold increased risk of LOS diagnosis in the next 24 h compared to the baseline risk of 0.25% in any given 24 h period during the NICU stay. The POWS model evaluated in this study on NEC is the same model previously developed for LOS.

### Statistical analysis

We used Wilcoxon rank sum tests and chi-squared tests to compare continuous and categorical clinical variables among infants with LOS, NEC, both diagnoses (non-concurrent), or neither diagnosis.

We explored the relationships between individual cardiorespiratory features and LOS and NEC using univariable logistic regression models. We plotted the log odds of event risk to visualize ranges of cardiorespiratory features associated with significantly increased or decreased risk of LOS or NEC. To account for repeated hourly measures, we corrected for unequal variances and correlated measures from individual patients. The confidence intervals in the univariable figures were adjusted for repeated measures using the Huber-White method to modify the variance-covariance matrix in all logistic regression models.^12^

To evaluate POWS performance in predicting NEC events relative to sepsis events, we calculated the area under the receiver operating characteristic (AUC) with confidence intervals based on 200 bootstrap runs resampled by admission. The POWS model, described above, was trained using all episodes of LOS and NEC with concurrent bacteremia in VLBW infants at UVA from 2012 to 2021. The AUCs reported here are calculated across external and within sample data with the aim of making comparisons of relative discrimination among events types. We compared hourly POWS scores for medical and surgical NEC using rank sum tests. Paired signed rank tests were used to analyze the rise in the POWS score before NEC diagnosis. Statistical analyses were performed in R 4.2.3 (R Foundation for Statistical Computing, Vienna, Austria) using the *rms* package.^12^

## Results

### Characteristics of infants with no event versus those with LOS, NEC, or both

We analyzed clinical data from 3914 VLBW infants at four NICUs. Of these, 515 (13%) had at least one episode of LOS and 210 (5%) had NEC (Table 1).

**Table 1:** Clinical characteristics of infants with and without LOS or NEC

Characteristic	No Event N = 3232^b^	Sepsis N = 472^b^	NEC N = 167^b^	Sepsis and NEC N = 43^b^
Gestational Age^a^ (weeks)	28.1 (26.0, 30.0)	25.4 (24.1, 27.3)	26.6 (25.1, 28.0)	24.9 (24.0, 26.9)
Birth Weight^a^ (g)	1040 (785, 1280)	750 (609, 923)	800 (630, 1030)	660 (590, 819)
Sex				
Female	1684 (52%)	217 (46%)	75 (45%)	20 (47%)
Male	1548 (48%)	255 (54%)	92 (55%)	23 (53%)
Race				
White	1738 (54%)	269 (57%)	89 (53%)	21 (49%)
Black	1266 (39%)	178 (38%)	73 (44%)	19 (44%)
Asian	96 (3.0%)	5 (1.1%)	1 (0.6%)	0 (0%)
Unknown	132 (4.1%)	20 (4.2%)	4 (2.4%)	3 (7.0%)
Died^a^	191 (5.9%)	79 (17%)	37 (22%)	11 (26%)

*LOS* late onset sepsis, *NEC* necrotizing enterocolitis.

^a^*p* < 0.05.

^b^Median (Q1, Q3); *n* (%).

Infants with LOS and/or NEC had lower mean GA and BW compared to those with neither diagnosis, and infants with LOS had lower mean GA compared to those with NEC. SGA status was not significantly different between groups among infants with GA < 30 weeks. The median age at the first episode of LOS was 17 days (IQR 8–32) and the median age at NEC diagnosis was 23 days (IQR 14–37) (*p* < 0.001). For the 43 infants with both NEC and non-concurrent LOS, NEC occurred first in 16 cases (37%).

Organisms causing LOS were Gram-negative bacteria in 101 cases, coagulase-negative staphylococcus (CONS) in 294, other Gram-positive bacteria in 113, and fungi in 7. In cases of NEC, 46 infants (22%) had a concurrent positive blood culture, with Gram-negative bacteria in 16 cases, CONS in 22, other Gram-positive bacteria in 7, and fungi in 1.

Of the 210 infants with NEC, 133 cases were treated medically and 77 cases required surgical management. Supplementary Table 1 shows characteristics of infants with medical versus surgical NEC. Those with surgical NEC had higher mortality (*p* < 0.001).

### Cardiorespiratory features in LOS and NEC

In total, 3110 (79%) infants with clinical data had continuous vital signs data available for analysis. Infants with available vital sign data were of significantly higher birthweight (990 grams, IQR 745–1245) than those infants with missing vital sign data (939 grams, IQR 700–1240) (*p* < 0.019). Gestational age, sex, and rate of survival were not significantly different between the groups (Table S2). We also analyzed missing data by site (Table S3). We analyzed 3.4 million patient-hours of vital sign data from the 3110 VLBW infants, including around the time of 341/515 (66%) cases of LOS and 134/210 (64%) cases of NEC.

**Fig. 1:**
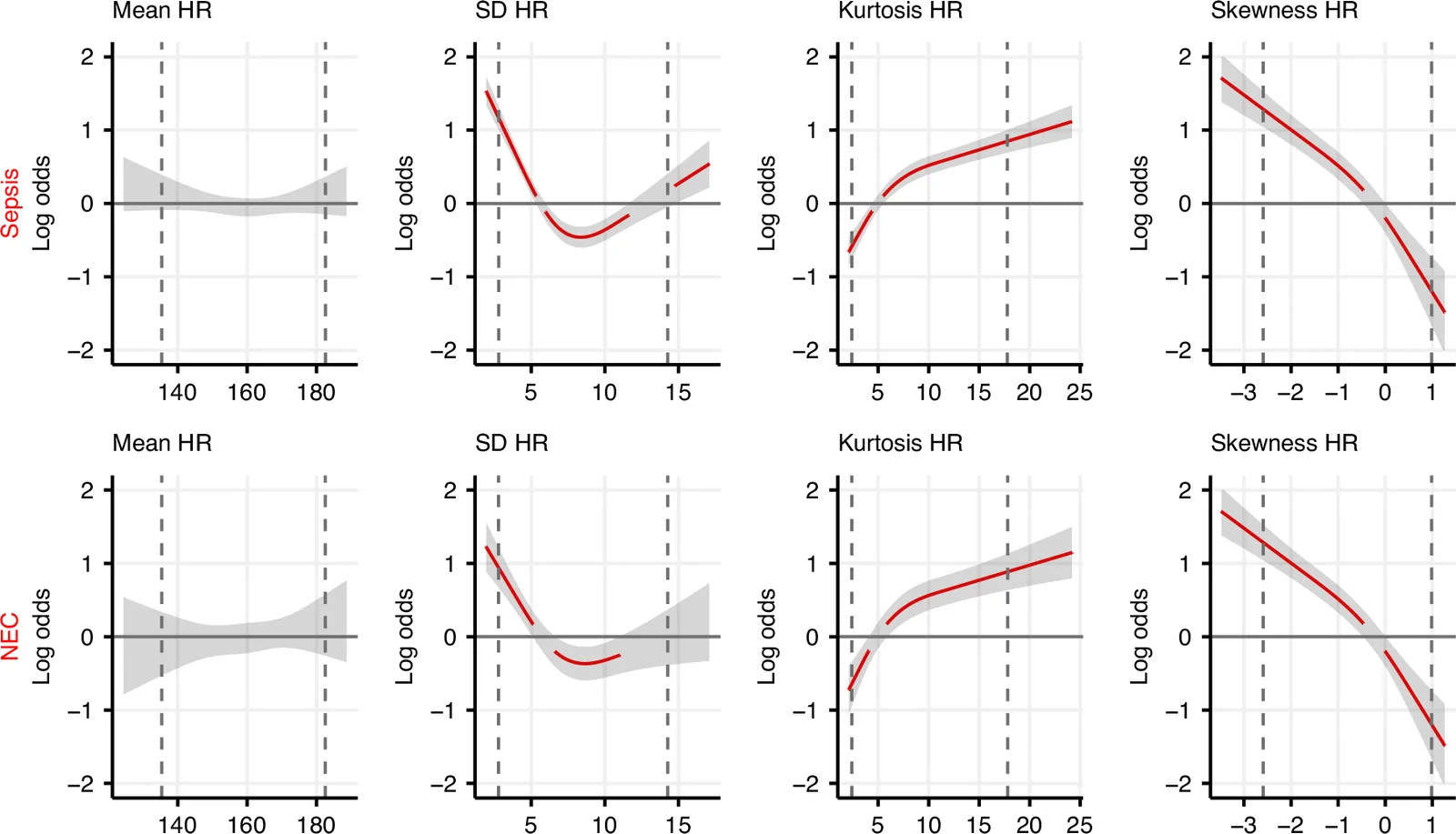
Sepsis and NEC risk based on heart rate features. Depiction of associations of heart rate features in each univariable logistic regression model for sepsis risk (top row) and NEC risk (bottom row). Each tile is a plot of the log odds of sepsis or NEC within the next 24 h as a function of the feature across its range, where 0 on the y-axis indicates risk equal to overall event risk for all VLBWs at all times. The translucent ribbon represents the 95% confidence interval, and the vertical dashed lines the 2.5 and 97.5 percentile of the data. The red line highlights the range where the confidence intervals do not include 0 and the variable may be considered a predictor of significantly increased or decreased event risk (above or below zero odds, respectively). The major finding is that the relationships between HR features and the log odds of event risk were similar for LOS and NEC.

**Fig. 2:**
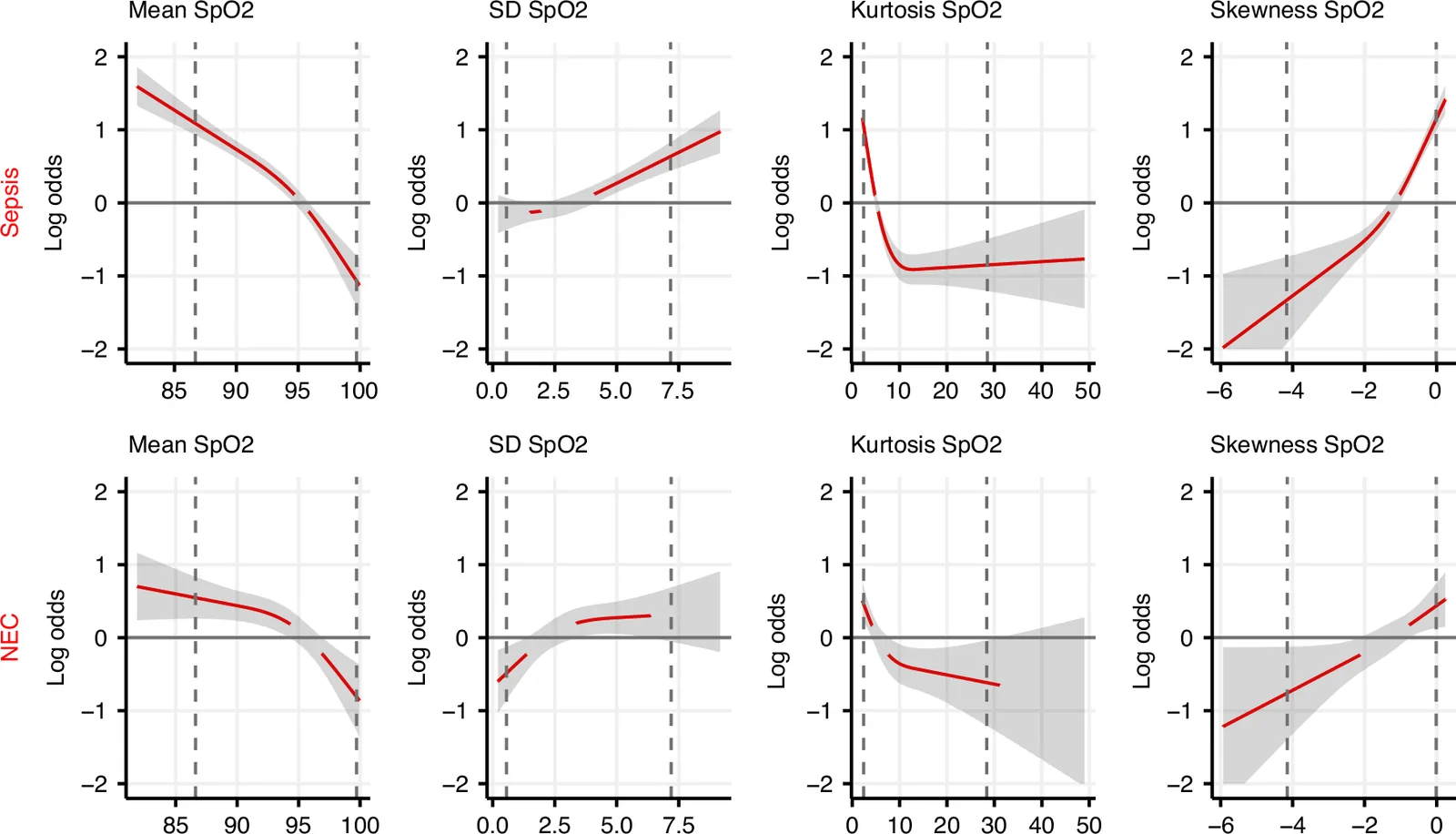
Sepsis and NEC risk based on SpO_2_ features. Depiction of associations of SpO_2_ features in each univariable logistic regression model for sepsis risk (top row) and NEC risk (bottom row). A description of the plot features is in Fig. 1 legend. The major finding is that the relationships between SpO_2_ features and the log odds of event risk were similar for LOS and NEC.

**Fig. 3:**
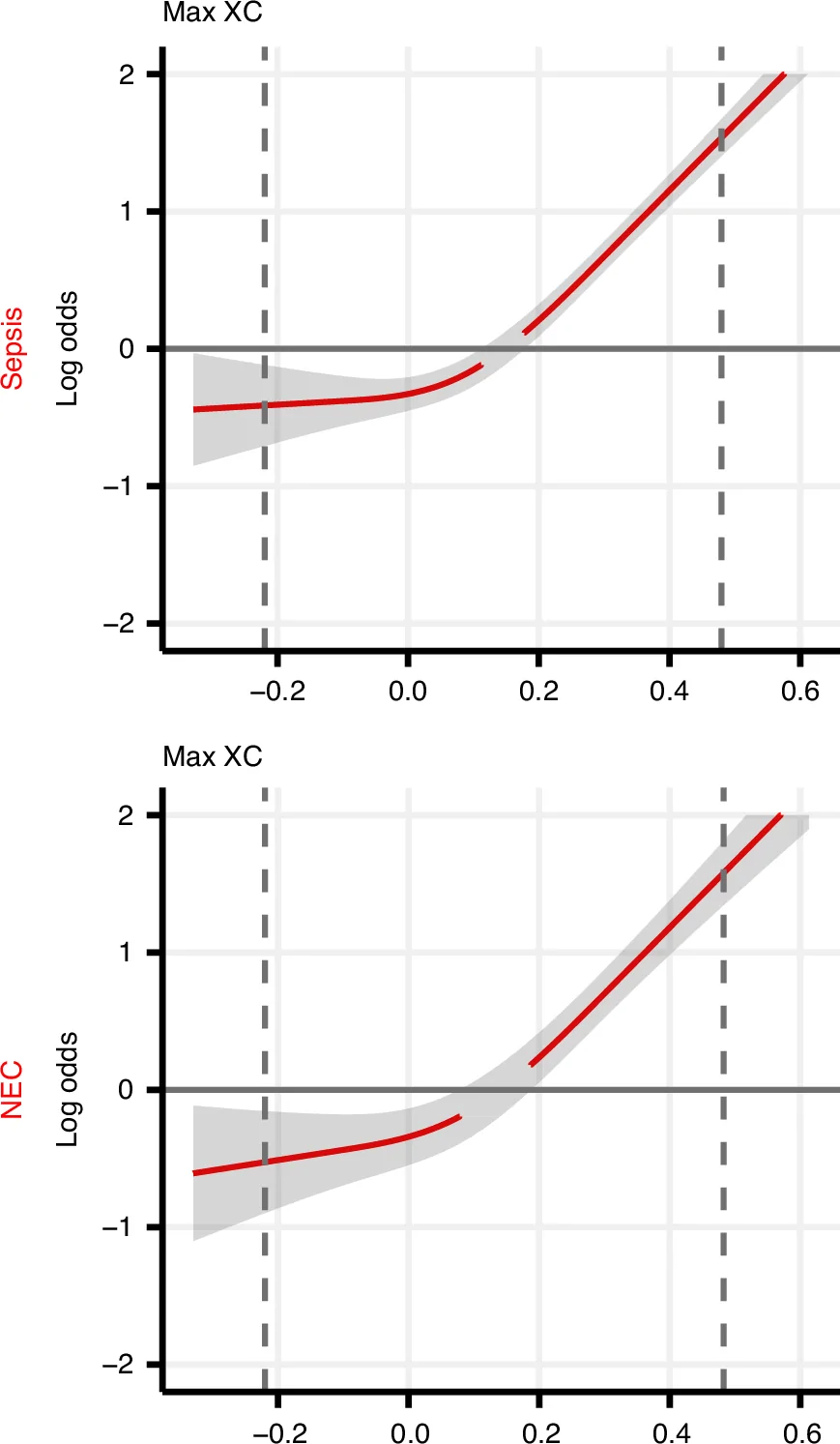
Sepsis and NEC risk based on cross correlation of HR-SpO_2_. Associations of maximum XCORR-HR-SpO_2_ and risk of imminent sepsis (top) and NEC (bottom). A description of the plot features is in Fig. 1 legend. The major finding is that the relationship between XCORR-HR-SpO_2_ and the log odds of event risk was similar for LOS and NEC.

Figures 1, 2, and 3 depict the results of univariable logistic regression models as the log odds of an event (LOS or NEC) in the subsequent 24 h for HR, SpO_2_, and XCORR-HR-SpO_2_ metrics, respectively. All features except mean HR were associated with either significantly increased or decreased event risk, and the relationship between HR and SpO_2_ features and the log odds of event risk were similar for LOS and NEC events. Generally, for HR, lower standard deviation (low variability) and more negative skewness (more decelerations) were associated with increased risk of an event in the next 24 h. For SpO_2_, lower mean and more negative skewness (more desaturations) were associated with higher risk of an imminent event. For XCORR-HR-SpO_2_, a higher value (indicating greater co-trending of the two values) was associated with higher risk for LOS or NEC diagnosis in the ensuing 24-hour period.

### POWS score AUC for imminent NEC or LOS

Table 2 shows the AUCs for POWS discriminating between infants in the 24 h preceding the event and all other times and infants without an event. AUC was highest for surgical NEC (0.808) and lowest for medical NEC (0.734).

**Table 2:** Discrimination of POWS for predicting sepsis or NEC within 24 h.

Outcome	Number of events	AUC (95% CI)
Sepsis or NEC	475	0.791 (0.791–0.792)
Sepsis	341	0.804 (0.804–0.806)
NEC (medical and surgical)	134	0.758 (0.756–0.760)
Surgical NEC	48	0.808 (0.805–0.812)
Medical NEC	86	0.734 (0.730–0.738)

*AUC* area under the receiver operating characteristic curve, *NEC* necrotizing enterocolitis, *CI* confidence interval.

### POWS score around the time of LOS and medical and surgical NEC

The average POWS score was significantly higher in the 24 h preceding a NEC or sepsis event (2.3 [IQR: 1.1–4.3) than at all other times (0.5 [IQR: 0.2–1.3) (*p* < 0.001), The average POWS score was not significantly higher in the 24 h preceding LOS (2.4 [IQR: 1.2–4.5]) compared to NEC (2.0 [IQR: 0.8–3.7]) (*p* = 0.067). The POWS score in infants with NEC and concurrent bacteremia was higher [3.3 (IQR 1.6–5.4)] than those with NEC and a negative blood culture [1.7 (IQR 0.7–3.2)] (*p* = 0.019).

**Fig. 4:**
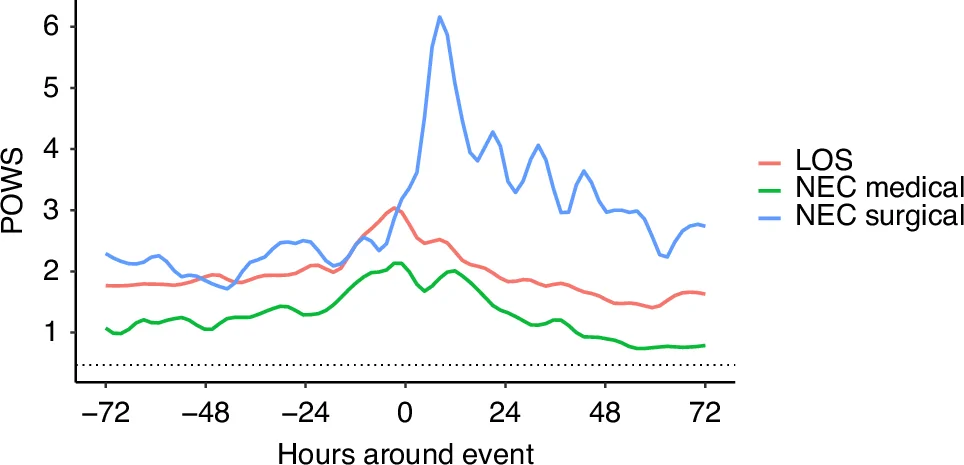
Pulse Oximetry Warning Score in very low birth weight infants with medical or surgical NEC or late onset sepsis. 134 VLBW infants with NEC and 341 with LOS had vital sign data available around the time of diagnosis. POWS incorporates 7 HR, SpO_2_, and XCORR metrics and is shown for 6 days surrounding diagnosis of LOS (red *n* = 341), medical NEC (green, *n* = 86) and NEC requiring surgery (blue, *n* = 48). The dashed horizontal line represents the median POWS score for all VLBW infants throughout the NICU stay.

In the hours preceding diagnosis of both medical and surgical NEC, there was a rise in the mean POWS score over infants’ prior 24-h baseline (Fig. 4). A statistically significant rise from baseline occurred 1 h before diagnosis of medical NEC and 11 h before diagnosis of surgical NEC. At the time of diagnosis, patients with surgical NEC had a higher median POWS score (3.5[IQR 1.7–5.8]) compared to those with medical NEC (2.4 [IQR 0.8–4.3]) (*p* = 0.014). Examining POWS score by time to surgery, those infants having surgery within 48 h had a similar POWS score prior to diagnosis compared to those with surgery more than 48 h after diagnosis (Fig. S1).

## Discussion

Detecting NEC in the early stages of illness, prior to obvious clinical deterioration, will lead to earlier treatment and may improve outcomes. We found that a continuously calculated POWS (Pulse Oximetry Warning System) score, previously developed for LOS prediction in VLBW infants, also rises in the preclinical phase of NEC, regardless of concurrent bacteremia. The dynamic rise in risk demonstrated by POWS was more prominent for cases of surgical NEC compared with medical NEC, even when surgery occurred remote from clinical diagnosis.

Decades ago, our group developed a heart rate characteristics (HRC) index (HeRO score) as an early warning system for LOS, incorporating measures of low variability and repetitive transient heart rate decelerations.^13^ This pathologic pattern occurs in the preclinical phase of sepsis, before overt clinical deterioration, and is reminiscent of the fetal heart rate pattern of hypoxia-ischemia or fetal compromise associated with chorioamnionitis.^14^ Display of the HRC index, without a mandate for intervention, was associated with a 40% decrease in sepsis-associated mortality in a randomized trial of 3003 VLBW infants at 9 NICUs.^15,16^ This electrocardiogram-based score also rises hours before clinical diagnosis of NEC.^6^ In addition to altering HR characteristics, acute systemic inflammatory illnesses can lead to increased apnea and hypoxemia in premature infants, in part due to release of mediators including prostaglandins and cytokines that impact control of breathing.^17–20^ POWS captures shifts in the distribution and variability of SpO_2_ patterns as well as cross-correlation of HR and SpO_2_. High XCORR values in part represent increased apnea or exaggerated periodic breathing with associated deceleration-desaturation.^11^ In a two-NICU study, we reported that XCORR-HR-SpO_2_, using a lag time of 30 seconds between the two signals, provided the greatest discrimination for clinical deterioration prior to diagnosis of NEC or LOS from a group of candidate cardiorespiratory metrics.^10^ In that study, predictive model performance was better for infants not on mechanical ventilation at the time of illness, which aligns with the metric being sensitive to apnea or pauses in breathing. Our research has expanded to four NICUs, and in the current work, we found commonality in the cardiorespiratory signatures of NEC and LOS, reflecting the overlapping physiology of these inflammatory illnesses. In cases of surgical NEC, POWS rose more steeply and peaked higher, potentially reflecting an even more robust inflammatory response accompanying intestinal necrosis, perforation, and peritonitis. Even among infants for whom surgery occurred more than 48 h after NEC diagnosis, POWS increased higher than in infants with medical NEC, indicating that the more significant rise was not due to surgery alone but related to greater severity of illness.

Other groups have studied vital sign patterns prior to NEC. Depressed heart rate variability (HRV) was noted as much as 2 days prior to diagnosis of NEC in a study of 30 preterm NICU patients.^21^ In that study, the magnitude of HRV change was associated with severity of NEC, and HRV returned to baseline 2 days after diagnosis. Adding more predictors could improve model performance, and other groups have developed prediction models for LOS and NEC that include additional vital signs, laboratory values, and clinical signs or events.^22,23^ Some NEC models include both continuous vital signs and clinical variables, while others focus solely on variables available from the electronic health record (EHR) including hourly or less frequently sampled vital signs.^22–24^ We chose to include only continuous HR and SpO2 data in our model, as these data contain the earliest and least biased signs of change in physiology due to infection or inflammation. This type of early warning system is designed to trigger a clinical evaluation that leads to diagnosis rather than to make the diagnosis. Laboratory values, intermittent assessments, medications, and other EHR data can increase model specificity for diagnosis, but represent provider behavior or activity and are inherently reactive to clinical concerns. We also do not include static variables such as demographics in our model, as these can provide risk stratification but do not provide early warning of clinical deterioration.

While VLBW infants admitted to the NICU are generally at risk for adverse events and clinical decompensation, most are not critically ill at any given time, making them an ideal population for vital sign-based predictive analytics. Our work has focused, for the most part, on identifying changes in cardiorespiratory metrics as NICU patients transition from “not sick” (or chronically sick) to “acutely sick” over short time windows ( < 24 h prior to event). Another risk prediction approach is to identify cardiorespiratory patterns in the first days or weeks after birth that predict adverse outcomes farther into the future. Studies of early heart rate variability have had mixed results in the ability to predict the risk of later diagnosis of sepsis or NEC.^21,25^ Some have reported that low high-frequency HRV, representing low parasympathetic (vagal) tone, may be associated with the development of NEC as much as several weeks later.^26^ Further research into the interactions between the three divisions of the autonomic nervous system (enteric, sympathetic, and parasympathetic) is warranted to understand prognostic and therapeutic approaches to gastrointestinal diseases, including NEC.^27,28^

A strength of this study is a larger and multi-NICU cohort compared to other studies evaluating predictive models for NEC and LOS, but we acknowledge limitations as well. Some infants and events were excluded due to missing vital sign data and, given the retrospective nature of the study, we are not able to evaluate every clinical variable that may impact vital sign patterns, such as medications, cardiorespiratory diagnoses, and level of respiratory support. We also note that a rising POWS score can represent non-infectious inflammatory conditions such as acute exacerbation of chronic lung disease,^29^ which highlights the importance of considering all clinical variables before making a decision on whether to test and treat, or wait and watch closely.

The use of machine learning to detect signatures of illness and inform clinical action has great potential for benefit in the NICU.^30^ Our analysis of a Pulse Oximetry Warning Score (POWS) provides a foundation for implementation in a clinical trial setting. Further evaluation is necessary to understand the impact POWS will have on clinical care, including risks and benefits.

## Conclusions

A Pulse Oximetry Warning Score (POWS) designed as a continuous risk indicator of imminent sepsis also rises in the preclinical phase of NEC. Use of this physiomarker could alert clinicians to NICU patients requiring further evaluation, potentially leading to earlier treatment of life-threatening illnesses. These results will inform the next steps towards implementation of POWS, such as prospective evaluation and clinical trials.

## Supplementary information


Appendix resubmission


## Data Availability

The datasets generated during and/or analyzed during the current study are available from University of Virginia Dataverse.

## References

[1] Patel, A. L., Panagos, P. G. & Silvestri, J. M. Reducing incidence of necrotizing enterocolitis. *Clin. Perinatol.* **44**, 683–700 (2017). 28802346 10.1016/j.clp.2017.05.004

[2] Garg, P. M. et al. Clinical impact of NEC-associated sepsis on outcomes in preterm infants. *Pediatr. Res.* **92**, 1705–1715 (2022). 35352003 10.1038/s41390-022-02034-7 PMC10311923

[3] Rees, C. M., Pierro, A. & Eaton, S. Neurodevelopmental outcomes of neonates with medically and surgically treated necrotizing enterocolitis. *Arch. Dis. Child Fetal Neonatal. Ed.* **92**, F193–F198 (2007). 16984980 10.1136/adc.2006.099929 PMC2675329

[4] Garg, P. M. et al. Severe acute kidney injury in neonates with necrotizing enterocolitis: risk factors and outcomes. *Pediatr. Res.* **90**, 642–649 (2021). 33446918 10.1038/s41390-020-01320-6 PMC8277891

[5] Griffin, M. P., Lake, D. E., O’Shea, T. M. & Moorman, J. R. Heart rate characteristics and clinical signs in neonatal sepsis. *Pediatr. Res.* **61**, 222–227 (2007). 17237726 10.1203/01.pdr.0000252438.65759.af

[6] Stone, M. L. et al. Abnormal heart rate characteristics before clinical diagnosis of necrotizing enterocolitis. *J. Perinatol.* **33**, 847–850 (2013). 23722974 10.1038/jp.2013.63 PMC4026091

[7] Sullivan, B. A. et al. Clinical and vital sign changes associated with late-onset sepsis in very low birth weight infants at 3 NICUs. *J. Neonatal Perinat. Med.* **14**, 553–561 (2021). 10.3233/NPM-200578 PMC8316489 33523025

[8] Kausch, S. L. et al. Cardiorespiratory signature of neonatal sepsis: development and validation of prediction models in 3 NICUs. *Pediatr. Res.* **93**, 1913–1921 (2023). 36593281 10.1038/s41390-022-02444-7 PMC10314957

[9] Bell, M. J. et al. Neonatal necrotizing enterocolitis. Therapeutic decisions based upon clinical staging. *Ann. Surg.* **187**, 1–7 (1978). 413500 10.1097/00000658-197801000-00001 PMC1396409

[10] Fairchild, K. D. et al. Vital signs and their cross-correlation in sepsis and NEC: a study of 1,065 very-low-birth-weight infants in two NICUs. *Pediatr. Res.* **81**, 315–321 (2017). 28001143 10.1038/pr.2016.215 PMC5309159

[11] Fairchild, K. D. & Lake, D. E. Cross-correlation of heart rate and oxygen saturation in very low birthweight infants: association with apnea and adverse events. *Am. J. Perinatol.* **35**, 463–469 (2018). 29141263 10.1055/s-0037-1608709 PMC6543270

[12] Harrell F. *Regression modeling strategies: with applications to linear models, logistic and ordinal regression, and survival analysis* (Springer Series in Statistics, 2015).

[13] Lake, D. E., Griffin, M. P. & Moorman, J. R. New mathematical thinking about fetal heart rate characteristics. *Pediatr. Res.* **53**, 889–890 (2003). 12646723 10.1203/01.PDR.0000064907.53534.90

[14] Muraoka, J., Kodama, Y., Ohashi, M., Goto, T. & Sameshima, H. Intrapartum fetal heart rate patterns and perinatal outcome in chorioamnionitis at or beyond 34 weeks of gestation. *J. Obstet. Gynaecol. Res.* **47**, 1110–1117 (2021). 33403794 10.1111/jog.14641

[15] Moorman, J. R. et al. Mortality reduction by heart rate characteristic monitoring in very low birth weight neonates: a randomized trial. *J. Pediatr.* **159**, 900–6.e1 (2011). 21864846 10.1016/j.jpeds.2011.06.044 PMC3215822

[16] Fairchild, K. D. et al. Septicemia mortality reduction in neonates in a heart rate characteristics monitoring trial. *Pediatr. Res.* **74**, 570–575 (2013). 23942558 10.1038/pr.2013.136 PMC4026205

[17] Herlenius, E. An inflammatory pathway to apnea and autonomic dysregulation. *Respir. Physiol. Neurobiol.* **178**, 449–457 (2011). 21762793 10.1016/j.resp.2011.06.026

[18] Hofstetter, A. O., Saha, S., Siljehav, V., Jakobsson, P.-J. & Herlenius, E. The induced prostaglandin E2 pathway is a key regulator of the respiratory response to infection and hypoxia in neonates. *Proc. Natl. Acad. Sci. USA* **104**, 9894–9899 (2007). 17535900 10.1073/pnas.0611468104 PMC1877988

[19] Siljehav, V., Hofstetter, A. M., Leifsdottir, K. & Herlenius, E. Prostaglandin E2 mediates cardiorespiratory disturbances during infection in neonates. *J. Pediatr.* **167**, 1207–13.e3 (2015). 26434370 10.1016/j.jpeds.2015.08.053

[20] Balan, K. V. et al. Vagal afferents modulate cytokine-mediated respiratory control at the neonatal medulla oblongata. *Respir. Physiol. Neurobiol.* **178**, 458–464 (2011). 21397055 10.1016/j.resp.2011.03.003 PMC3150618

[21] Al-Shargabi, T. et al. Changes in autonomic tone in premature infants developing necrotizing enterocolitis. *Am. J. Perinatol.* **35**, 1079–1086 (2018). 29609189 10.1055/s-0038-1639339

[22] Gephart, S. M. et al. Discrimination of GutCheck(NEC): a clinical risk index for necrotizing enterocolitis. *J. Perinatol.* **34**, 468–475 (2014). 24651734 10.1038/jp.2014.37 PMC4420242

[23] Mithal, L. B. et al. Vital signs analysis algorithm detects inflammatory response in premature infants with late onset sepsis and necrotizing enterocolitis. *Early Hum. Dev.* **117**, 83–89 (2018). 29351876 10.1016/j.earlhumdev.2018.01.008 PMC5983899

[24] Masino, A. J. et al. Machine learning models for early sepsis recognition in the neonatal intensive care unit using readily available electronic health record data. *PLoS ONE* **14**, e0212665 (2019). 30794638 10.1371/journal.pone.0212665 PMC6386402

[25] Sullivan, B. A. et al. Early pulse oximetry data improves prediction of death and adverse outcomes in a two-center cohort of very low birth weight infants. *Am J Perinatol.* **35**, 1331–1338 (2018). 10.1055/s-0038-1654712 PMC6262889 29807371

[26] Meister, A. L. et al. Vagal tone and proinflammatory cytokines predict feeding intolerance and necrotizing enterocolitis risk. *Adv. Neonatal Care* **21**, 452–461 (2021). 34847103 10.1097/ANC.0000000000000959 PMC8638911

[27] Ali M. K., Chen J. D. Z. Roles of heart rate variability in assessing autonomic nervous system in functional gastrointestinal disorders: a systematic review. *Diagnostics* **13**, 293 (2023). 10.3390/diagnostics13020293 PMC9857852 36673103

[28] Chandramowlishwaran, P., Raja, S., Maheshwari, A. & Srinivasan, S. Enteric nervous system in neonatal necrotizing enterocolitis. *Curr. Pediatr. Rev.* **18**, 9–24 (2022). 34503418 10.2174/1573396317666210908162745

[29] Kausch, S. L. et al. Clinical correlates of a high cardiorespiratory risk score for very low birth weight infants. *Pediatr. Res.* **97**, 2005–2009 (2024) 10.1038/s41390-024-03580-y PMC12122351 39300276

[30] Sullivan, B. A. et al. Transforming neonatal care with artificial intelligence: challenges, ethical consideration, and opportunities. *J. Perinatol.* **44**, 1–11 (2024). 38097685 10.1038/s41372-023-01848-5 PMC10872325

