# Supplementary material for: Cardiorespiratory signatures of necrotizing enterocolitis: a 4-NICU study of very low birth weight infants
Source: Pediatr Res. 2025 Dec 16;100(1):403–9. doi: 10.1038/s41390-025-04631-8 (PMC13042325; doi:10.1038/s41390-025-04631-8)
Supplement: Supplementary file 1 [file 41390_2025_4631_MOESM1_ESM.pdf]

Appendix

Table S1: Characteristics of infants with medical versus surgical NEC.

Characteristic	Medical NEC N = 133[1]	Surgical NEC N = 77[1]
Gestational Age (weeks)	26.6 (25.0, 28.0)	26.0 (24.4, 27.7)
Birth Weight (g)	791 (610, 1,050)	730 (630, 898)
Age at NEC	24 (16, 40)	23 (12, 35)
Sex		
Female	65 (49%)	30 (39%)
Male	68 (51%)	47 (61%)
Positive Blood Culture	26 (20%)	20 (26%)
Died*	15 (11%)	33 (43%)

[1] Median (Q1, Q3); n (%); * p-value < 0.001

Table S2. Characteristics of infants with HR and SpO2 data available for feature calculation

Characteristic	Overall N = 3,914[1]	Data Missing N = 804[1]	Data Available N = 3,110[1]	p-value[2]
Gestational Age (weeks)	27.9 (25.9, 30.0)	27.7 (25.9, 30.0)	27.9 (25.9, 30.0)	0.5
Birth Weight (g)	980 (730, 1,245)	939 (700, 1,240)	990 (745, 1,245)	0.019
Sex				0.082
Female	1,996 (51%)	432 (54%)	1,564 (50%)	
Male	1,918 (49%)	372 (46%)	1,546 (50%)	
Died	318 (8.1%)	75 (9.3%)	243 (7.8%)	0.2

[1] Median (Q1, Q3); n (%)

[2] Wilcoxon rank sum test; Pearson's Chi-squared test

Table S3. Characteristics of infants with HR and SpO2 data available for feature calculation by site

Characteristic	CU		UAB		UVA		WU	
	Data Missing N = 282[1]	Data Available N = 849[1]	Data Missing N = 266[1]	Data Available N = 456[1]	Data Missing N = 101[1]	Data Available N = 1,084[1]	Data Missing N = 155[1]	Data Available N = 721[1]
Gestational Age (weeks)	28.0 (26.0, 31.0)	29.0 (26.0, 31.0)	26.9 (25.4, 28.3)	26.9 (24.7, 28.6)	28.0 (25.7, 31.0)	28.1 (25.9, 30.3)	28.3 (26.3, 29.7)	27.3 (25.4, 29.1)
Birth Weight (g)	990 (725, 1,315)	1,090 (775, 1,315)	846 (630, 1,059)	842 (620, 1,090)	1,010 (690, 1,310)	1,020 (790, 1,263)	1,060 (800, 1,297)	940 (725, 1,180)
event								
No Event	248 (88%)	736 (87%)	228 (86%)	364 (80%)	84 (83%)	861 (79%)	139 (90%)	572 (79%)
Sepsis	14 (5.0%)	72 (8.5%)	27 (10%)	57 (13%)	10 (9.9%)	177 (16%)	9 (5.8%)	106 (15%)
NEC	17 (6.0%)	36 (4.2%)	11 (4.1%)	26 (5.7%)	4 (4.0%)	36 (3.3%)	4 (2.6%)	33 (4.6%)
Sepsis and NEC	3 (1.1%)	5 (0.6%)	0 (0%)	9 (2.0%)	3 (3.0%)	10 (0.9%)	3 (1.9%)	10 (1.4%)
Died	15 (5.3%)	40 (4.7%)	34 (13%)	63 (14%)	8 (7.9%)	43 (4.0%)	18 (12%)	97 (13%)

[1] Median (Q1, Q3); n (%)

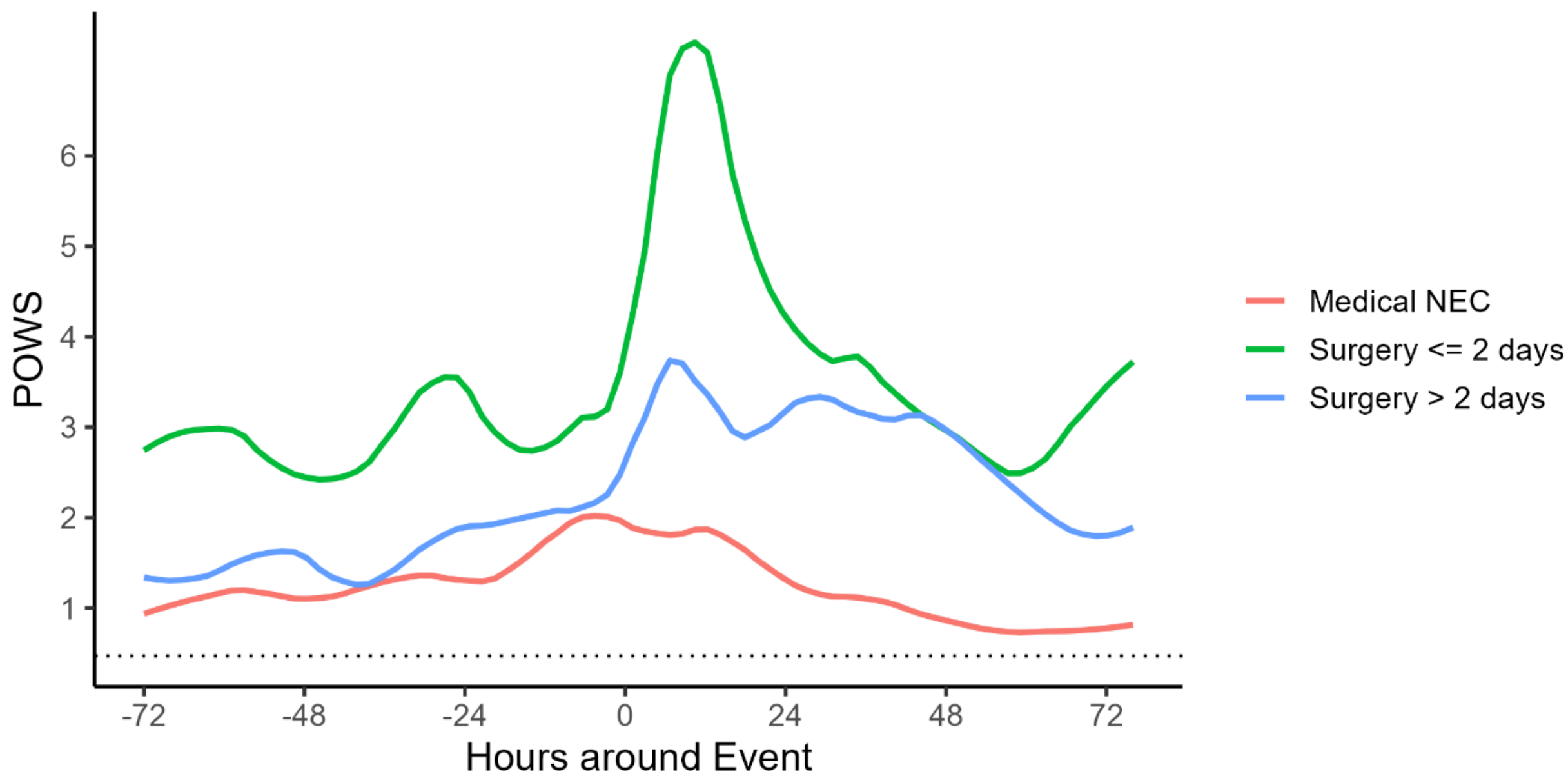

Figure S1. Pulse Oximetry Warning Score in very low birth weight infants with medical versus early and late surgical NEC. 134 VLBW infants with NEC had vital sign data available around the time of diagnosis. POWS is shown for 6 days surrounding diagnosis of medical NEC (red, n=86), NEC requiring surgery within 2 days of diagnosis (green, n=28), and NEC requiring surgery after 2 days (blue, n = 18). The dashed horizontal line represents the median POWS score for all VLBW infants throughout the NICU stay. Two infants excluded with unknown surgery date.
